# Smallpox History and Decisive Actions of George Washington

**DOI:** 10.3201/eid3210.AC3210

**Published:** 2026-10

**Authors:** Terence Chorba

**Affiliations:** Centers for Disease Control and Prevention, Atlanta, Georgia, USA

**Keywords:** smallpox, viruses, vaccines, George Washington, Gilbert Stuart, art–science connection

**Figure:**
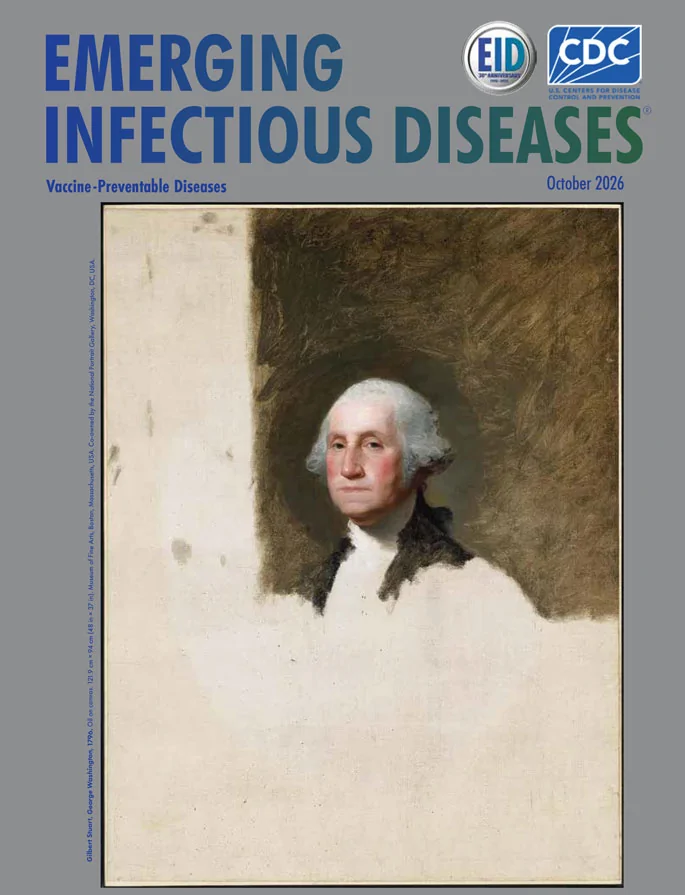
**Gilbert Stuart, *George Washington*,** 1796. Oil on canvas. 121.9 cm × 94 cm (48 in × 37 in). Museum of Fine Arts, Boston, Massachusetts, USA. Co-owned by the National Portrait Gallery, Washington, DC, USA.

As the United States celebrates its semiquincentennial year, the nation’s first president, George Washington, is a focal point. On the cover of this month’s *Emerging Infectious Diseases* is one of his best-known representations, the second Washington portrait that Gilbert Stuart (1755–1828) painted. This painting was commissioned by Washington’s wife, Martha, and begun in 1796. Stuart never wanted to part with this work and hence left it unfinished. This painting later became the template for the engraving of Washington on the US $1 bill.

A Rhode Islander by birth, Stuart had studied portraiture with Benjamin West in London, UK, pursued a brilliant career as a portraitist in Britain and Ireland, and returned to the United States in 1793. In addition to George Washington, he created portraits of Martha Washington (Figure), as well as John Jay, John Adams, Thomas Jefferson, John Quincy Adams, James Madison, and James Monroe, among others.

**Figure:**
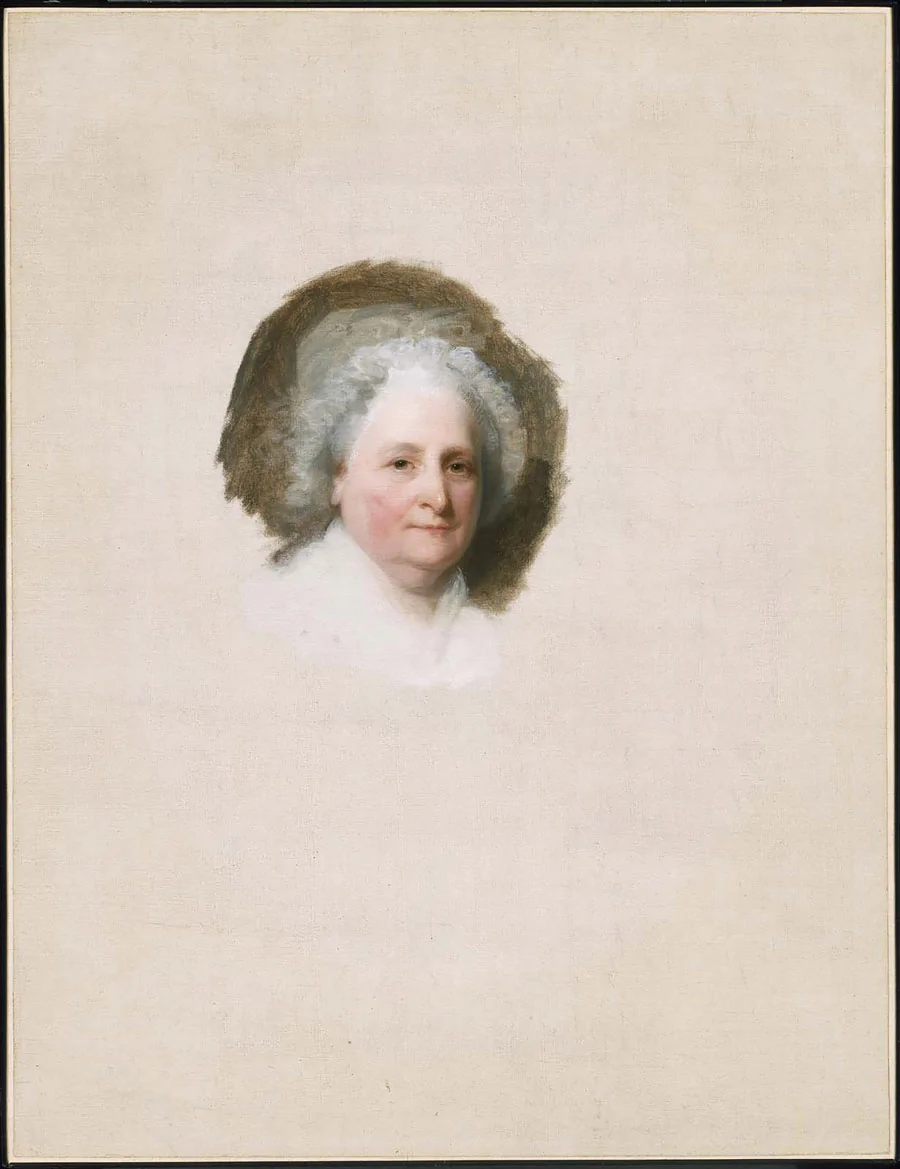
Gilbert Stuart, *Martha Washington*, 1796 (detail). Oil on canvas. 121.9 cm × 94.3 cm (48 in × 37 in). National Portrait Gallery, Washington, DC, USA. Co-owned by the Museum of Fine Arts, Boston, Massachusetts, USA.

In 1751, Lawrence Washington, George’s elder half-brother, was suffering from tuberculosis and had George accompany him to Barbados as a potential refuge. During that stay, the younger Washington contracted smallpox, enduring an illness lasting several weeks with residual minor facial scarring (*1*). Although the cover painting and others of Washington by Stuart are devoid of smallpox scars, at least one less-idealized Washington portrait rendered by William Joseph Williams from 1794 shows residual pockmarks (*2*). Washington’s firsthand familiarity with the progression and consequences of smallpox would later inform his decisions related to the health of the troops, demonstrated in several pieces of correspondence cited in this essay. In a set of general orders written in March 1776 in Boston, Washington observed that “as the enemy with a malicious assiduity, have spread the infection of the smallpox through all parts of the town, nothing but the utmost caution on our part, can prevent that fatal disease from spreading thro’ the army, and country, to the infinite detriment of both” (*3*).

As a preventive measure for smallpox, variolation was first promoted in the New World by Puritan minister Cotton Mather in 1721 (*4*). The procedure entailed insertion or rubbing of infectious material from pustules of persons infected with smallpox into the skin of persons who had not previously been infected. That process usually resulted in infection with mild symptoms, but also carried risk for potential spread of infection to others and for severe disease or death (*5*). However, variolation had a much lower associated case-fatality rate than that of naturally acquired smallpox, which was usually 25%–30% (*6*). In a well-documented epidemic in Boston, Massachusetts, in 1752, the reported mortality rate for those with natural smallpox was an unusually low 9%, but the rate was only 1.5% among those who underwent variolation (*4*). In a Charleston, South Carolina, epidemic in 1760, the mortality rate for natural smallpox cases was 34% but only 2.6% among those variolated (*4*).

Washington’s evolving stance on variolation reflected consideration of and respect for medical innovation, its attendant hazards, and military necessity. In 1776, as Commander-in-Chief of the Continental Army, he initially prohibited routine variolation. His reasoning was strategic: some soldiers undergoing variolation would be incapacitated for weeks, potentially leaving the army vulnerable at a critical juncture in the war. He observed, “… should We innoculate generally, the Enemy, knowing it, will certainly take Advantage of our Situation” (*7*). Given the precarious position of the colonial forces, the temporary loss of short-term operational readiness was a risk (*4*). However, smallpox outbreaks threatened losses, and Washington reversed his earlier policy. In February 1777, he ordered the systematic inoculation (i.e., variolation) of Continental troops:

Finding the Small pox to be spreading… I have determined that the troops shall be inoculated…. Necessity not only authorizes but seems to require the measure, for should the disorder infect the Army in the natural way and rage with its usual virulence we should have more to dread from it than from the Sword of the Enemy (*8*).

In correspondence with John Hancock, Washington justified the decision as necessary, despite its inherent drawbacks: “… I find it impossible to keep it from spreading thro’ the whole Army in the natural way. I have therefore determined, not only to innoculate all the Troops now here, that have not had it, but… to innoculate the Recruits as fast as they come in…” (*9*). New recruits would be inoculated upon arrival, recover while being trained and equipped, and then join the ranks immunized and combat-ready.

Elizabeth Fenn, author of *Pox Americana: The Great Smallpox Epidemic of 1775–82*, has noted that, in opting for universal variolation of the troops, “the general had outflanked his enemy” (*10*). By the end of 1777, tens of thousands of soldiers had undergone inoculation. The policy was an effective, organized public health intervention, demonstrating the capacity of centralized authority to manage disease on a large scale (*4*).

In 1796, English physician Edward Jenner identified a safer alternative. Observing that milkmaids who had contracted cowpox appeared resistant to smallpox, Jenner hypothesized that exposure to cowpox could protect against smallpox. His experimental inoculation of a young boy with cowpox material—and the boy’s subsequent immunity to smallpox—provided compelling evidence for vaccination (*11*). Inoculations with extracts from cowpox lesions (vaccination) or from horsepox lesions (equination) were safer and averted the onward transmission of smallpox. Vaccination succeeded because the species *Cowpox virus* and *Variola virus* (i.e., smallpox) belong to the same family (Poxviridae) and genus (*Orthopoxvirus*) of viruses, and some *Orthopoxvirus* species generate cross-immunity against infection with others.

In summary, smallpox played a central role in the American Revolution, posing what was perhaps a greater threat than the British to the Continental Army. Washington’s variolation program represented not merely a public health decision but a strategic military innovation with far-reaching consequences. That measure established an early precedent for government-led, population-level public health intervention. The intersection of Washington’s disease understanding, personal experience, and military leadership resulted in decisive implementation of immunization before there was vaccination; its impact was in the interest of public health and disease control and to the great advantage of the troops.

## Disclosure

The author discloses using ChatGPT (OpenAI, https://openai.com) only for locating references for this essay, not in generating content.

## References

[1] Chernow R. Washington: a life. New York: Penguin Press; 2010.

[2] Williams WJ. Portrait of George Washington in Masonic regalia, 1794. Wikimedia Commons [cited 2026 Sep 10]. https://commons.wikimedia.org/wiki/File:Portrait_of_George_Washington_in_Masonic_regalia_by_William_Joseph_Williams,_1794.jpg

[3] Washington G. General orders,13 March 1776. Founders Online, National Archives [cited 2026 Sep 10]. https://founders.archives.gov/documents/Washington/03-03-02-0336

[4] Wehrman A. The contagion of liberty: the politics of smallpox in the American Revolution. Baltimore (MD): Johns Hopkins University Press; 2022.

[5] Hopkins DR. The greatest killer: smallpox in history. Chicago: University of Chicago Press; 2002.

[6] Weiss RA, Esparza J. The prevention and eradication of smallpox: a commentary on Sloane (1755) ‘An account of inoculation’. Philos Trans R Soc Lond B Biol Sci. 2015;370:20140378. 10.1098/rstb.2014.0378 25750241 PMC4360126

[7] Washington G. To Major General Horatio Gates, 5–6 February 1777. Founders Online, National Archives [cited 2026 Sep 10]. https://founders.archives.gov/documents/Washington/03-08-02-0267

[8] Washington G. To William Shippen Jr., 6 February 1777. Founders Online, National Archives [cited 2026 Sep 10]. https://founders.archives.gov/documents/Washington/03-08-02-0281

[9] Washington G. To John Hancock, 5 February 1777. Founders Online, National Archives [cited 2026 Sep 10]. https://founders.archives.gov/documents/Washington/03-08-02-0268

[10] Fenn EA. Pox Americana: the great smallpox epidemic of 1775–82. New York: Hill and Wang; 2001. 12166479

[11] Jenner E. An inquiry into the causes and effects of the variolae vaccinae. London: Samson Low; 1798.

